# Supplementary material for: A Feasible Way to Produce Carbon Nanofiber by Electrospinning from Sugarcane Bagasse
Source: Polymers (Basel). 2019 Nov 29;11(12):1968. doi: 10.3390/polym11121968 (PMC6960696; doi:10.3390/polym11121968)
Supplement: Supplementary file 1 [file polymers-11-01968-s001.pdf]

Supporting Information

A Feasible Way to Produce Carbon Nanofiber by Electrospinning from Sugarcane Bagasse

Wei Chen [1], Xin-Tong Meng [1], Hui-Hui Wang [1], Xue-Qin Zhang [1,2], Yi Wei [1], Zeng-Yong Li [1], Di Li [1], Ai-Ping Zhang [3], and Chuan-Fu Liu [1,*]

[1] State Key Laboratory of Pulp and Paper Engineering, South China University of Technology, Guangzhou 510640, PR China; geogeo_chen@163.com (W.C.); mxt08205200@163.com (X.-T.M.); wang.huihui@mail.scut.edu.cn (H.-H.W.); wygift@163.com (Y.W.); 13609763220@163.com (Z.-Y.L.); lhnlg0503@126.com (D.L.);

[2] College of Light Industry and Food Science, Zhongkai University of Agriculture and Engineering, Guangzhou 510225, PR China; zhangxueqin0228@163.com (X.-Q.Z.);

[3] College of Forestry and Landscape Architecture, South China Agricultural University, Guangzhou 510642, PR China; aiping@scau.edu (A.-P.Z.)

* Correspondence: chfliu@scut.edu.cn; Tel.: +86-20-87113912 (C.-F.L.)

Content

FT-IR spectra of SCB and different SCB esters.…………………………………………………..…....Figure S1

NMR spectra of different SCB esters……………………………………………………………....Figure S2

SEM images of electrospun PAN nanofiber mats in different stages……………………..…....Figure S3

The TGA/DTG data and residue content of different samples………………………..…..…....Table S1

The characterization of graphite structure derived from XRD of different CNFs.….....…..…..…..…....Table S2

Number of pages: 8

Number of figures: 3

Number of Tables: 2

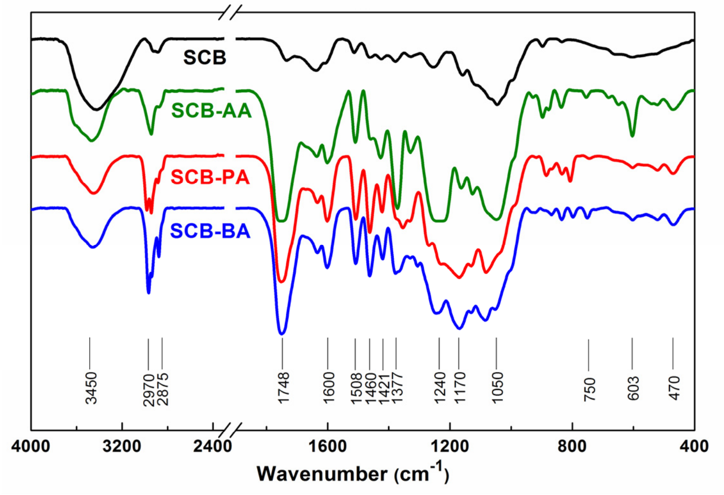

Figure S1. FT-IR spectra of SCB and different SCB esters.

In this study, the effect of chemical modification on the structure of SCB was demonstrated by FT-IR in the region of 4000–400 cm^{-1}. As shown in Figure S1, the intensity in broad region peak for -OH stretching vibrations at 3450 cm^{-1} of SCB-A showed an obvious decrease comparing to that of unmodified SCB. It indicated that the hydroxyl groups were involved in the homogeneous esterification [1]. In addition, the intensities of C–H bending vibrations (such as 1460, 1421 and 1377 cm^{-1}) were also enhanced after the introduction of aliphatic acyl groups. Meantime, a significant increase of peak intensity in the broad region at 1748 cm^{-1} for C=O stretching vibrations was observed. In general, these results indicated that the esterification of SCB with three acid anhydrides occurred successfully in the homogeneous system, which was consistent with the previous literatures [2-4].

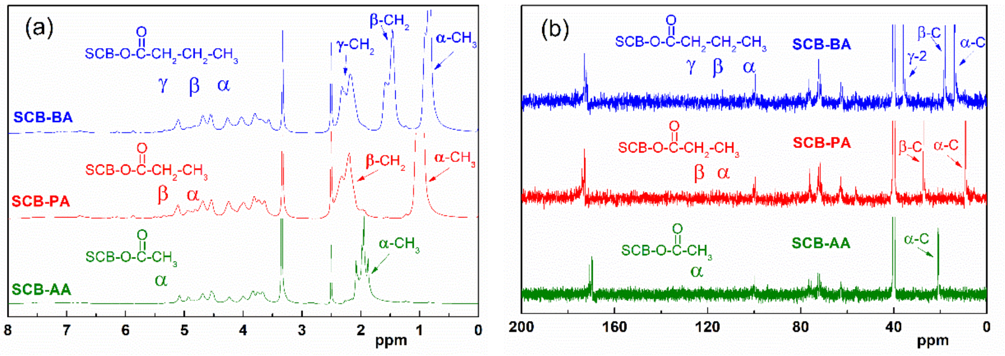

Figure S2. (**a**) ^{1}H NMR and (**b**) ^{13}C NMR spectra of different SCB esters.

To further confirm the esterification of SCB with acid anhydrides, SCB and SCB-A were characterized with ^{1}H NMR and ^{13}C NMR spectroscopy, and the spectra are shown in Figure S2. In the ^{1}H NMR spectra (Figure S2a), the signals at 2.5 and 3.3 ppm are assigned to DMSO-d_6 and water, respectively, and the signals in the range of 0.5 to 2.4 ppm are attributed to the acyl moieties [2,4]. In the ^{1}H NMR spectrum of SCB-AA, there was a wide signal between 1.6 and 2.2 ppm that was assigned to the hydrogen proton in methyl group (α-CH_3) from the attached acetyl group. The proton signals at 2.0-2.4 ppm and 0.5-1.2 ppm attributed to β-CH_2 and α-CH_3 of SCB-PA. In the ^{1}H NMR spectrum of SCB-BA, three distinct signals emerging in the chemical shift between 0.5 and 2.4 ppm corresponds to γ-CH_2 (δ_H=2.0-2.4 ppm), β-CH_2 (δ_H=1.2-1.7 ppm), and α-CH_3 (δ_H=0.5-1.0 ppm) groups [4]. The signals range from 173.0 to 169.0 ppm corresponds to the carbonyl of C=O were emerged in ^{13}C NMR spectrum of SCB-A (Figure S2b), which provided the direct evidence of the successful attachment of the corresponding aliphatic side chains onto SCB-A. The signal at 21.1 ppm is assigned to α-C of acetyl group in SCB-AA, the signals at 27.2 and 9.5 ppm are assigned to β-C and α-C of propionyl group in SCB-PA and the signals at 35.6, 18.2 and 13.9 ppm are assigned to the carbons of γ-C, β-C and α-C of butyryl group in SCB-BA, respectively. Those results confirmed the successful esterification of SCB, which was consistent with the results of FT-IR analysis above and other similar literatures [1,2,5].

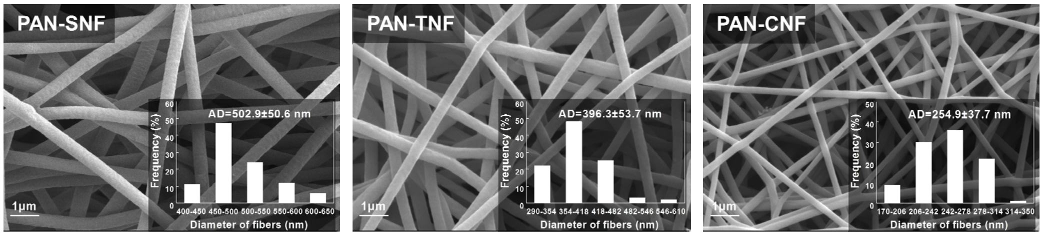

Figure S3. SEM images of electrospun PAN nanofiber mats in different stages (scale bar=1 μm).

Table S1. The TGA/DTG data and residue content of different samples

**Sample**	**T_{in} (°C)**[1]	**T_{50} (°C)**[2]	**T_{max} (°C)**[3]	**Residue (%)**[4]
SCB	275.8±1.7	332.2±3.1	345.5±6.7	16.1±7.0
SCB-AA	272.2±6.2	345.1±5.5	337.8±1.5	24.8±2.1
SCB-PA	281.9±2.6	355.5±2.1	356.1±2.9	17.6±4.8
SCB-BA	292.2±2.5	359.3±2.7	360.4±1.1	17.1±1.2
PAN-SNF	301.3±4.9	411.0±6.4	311.3±3.5	36.1±1.5
AA-SNF-50%	303.3±1.3	363.0±9.9	328.1±4.0	36.1±1.6
PA-SNF-50%	303.4±5.4	366.9±6.4	323.9±4.8	32.6±1.2
BA-SNF-50%	308.8±0.4	372.3±4.3	331.3±2.8	30.8±1.2

[1] T_{in}: Initial decomposition temperature; [2] T_{50}: 50% weight loss temperature; [3] T_{max}: temperature of the DTG peak; [4] Residue: the residue at 700 °C.

Table S2. The characterization of graphite structure derived from XRD of different CNFs

Sample	$d_{(002)}$ (Å)	Crystallite size, L_c (nm)	Lateral size, L_a (nm)
PAN-CNF	3.64±0.02	1.02±0.07	2.98±0.21
AA-CNF-50%	3.64±0.04	0.94±0.08	2.65±0.01
PA-CNF-50%	3.63±0.08	0.96±0.02	2.78±0.67
BA-CNF-50%	3.61±0.05	1.02±0.04	3.51±0.28

References

1. Xie, H.B.; King, A.; Kilpelainen, I.; Granstrom, M..Argyropoulos, D.S. Thorough Chemical Modification of Wood-Based Lignocellulosic Materials in Ionic Liquids. *Biomacromolecules* **2007**, 8, 3740-3748.
2. Zhang, X.Q.; Zhang, A.P.; Liu, C.F..Ren, J.L. Per-O-acylation of xylan at room temperature in dimethylsulfoxide/N-methylimidazole. *Cellulose* **2016**, 23, 2863-2876.
3. Chen, M.J.; Zhang, X.Q.; Liu, C.F..Shi, Q.S. Homogeneous Modification of Sugarcane Bagasse by Graft Copolymerization in Ionic Liquid for Oil Absorption Application. *Int. J. Polym. Sci.* **2016**, 3, 1-7.
4. Ding, R.; Wu, H.C.; Thunga, M.; Bowler, N..Kessler, M.R. Processing and characterization of low-cost electrospun carbon fibers from organosolv lignin/polyacrylonitrile blends. *Carbon* **2016**, 100, 126-136.
5. Wen, X.X.; Wang, H.H.; Wei, Y.; Wang, X.Y..Liu, C.F. Preparation and characterization of cellulose laurate ester by catalyzed transesterification. *Carbohydr. Polym.* **2017**, 168, 247-254.
